# A teleost structural analogue to the avian bursa of Fabricius

**DOI:** 10.1111/joa.13147

**Published:** 2019-12-26

**Authors:** Oskar M. Løken, Håvard Bjørgen, Ivar Hordvik, Erling O. Koppang

**Affiliations:** ^1^ Section of Anatomy Faculty of Veterinary Medicine Norwegian University of Life Sciences Oslo Norway; ^2^ Institute of Biology University of Bergen Bergen Norway

**Keywords:** adaptive immune system, bursa of Fabricius, evolutionary morphology, lymphoepithelium, lymphoid organs

## Abstract

The bursa of Fabricius is a primary and secondary lymphoid organ considered exclusively present in birds, and studies of this structure have been vital to our current understanding of the adaptive immune system of vertebrates. In this study, we reveal substantial lymphoepithelial tissue in a previously undescribed bursa in Atlantic salmon (*Salmo salar*), situated caudal to the urogenital papilla of the cloaca and thus analogous to the anatomical placement of the bursa of Fabricius. We investigated three groups of Atlantic salmon at different maturational stages and characterized the structure by applying dissection, radiology, scanning electron microscopy and histological techniques, including immunohistochemistry and *in situ* hybridization. We found that the epithelial anlage of the salmon cloacal bursa developed into substantial lymphoepithelial tissue and subsequently regressed following sexual maturation. Such a dynamic development is also a key characteristic of the avian bursa. The presence of intraepithelial lymphocytes was concomitant with expression of the leukocyte‐attracting chemokine CCL19, indicative of lymphoid organ functions. We did not observe recombination or gene conversion in salmon bursal lymphocytes at any developmental stage, indicating the absence of primary lymphoid organ functions in contrast to the bursa of Fabricius. However, the possibility of the bursa to trap both enteric and environmental antigens, combined with the presence of several antigen‐presenting cells residing within the lymphoepithelium, suggest the structure has secondary lymphoid organ functions. We present the discovery of a lymphoid organ in Atlantic salmon with striking topographical similarities to that of the bursa of Fabricius in birds. In addition, the age‐dependent dynamics of its lymphoepithelium suggest functions related to the maturation processes of lymphocytes.

## Graphical abstract

We have identified an undescribed lymphoepithelial compartment in the cloacal region of the Atlantic salmon with striking anatomical and developmental similarities to the bursa of Fabricius. This is the first report of such a structure in a non‐avian species. Our results raise the possibility of an earlier phylogenetic diversification of a lymphoid organ at this site than previously thought, or alternatively, a convergent evolutionary event.

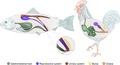

## Introduction

The bursa of Fabricius is a blind, lymphoepithelial diverticulum of ectodermal origin, located in the dorsal proctodeum of the avian cloaca (Nagy & Oláh, 2010). Studies of the bursa have been paramount to our understanding of the vertebrate adaptive immune system, because they revealed the divergence of two major lymphocyte lineages, T and B cells, with the latter obtaining its name from this structure (Cooper et al. 1966). As both a primary and secondary lymphoid organ, it is responsible for the diversification and maturation of avian B cells, which respond to alimentary and environmental antigens present in its lumen. The cloacal bursa is considered exclusive to birds; in mammals, functional analogues are found in other organs (Ekino & Sonoda, 2014). Consummate understanding of the adaptive immune system must include studies of the evolution and construction of primary and secondary lymphoid tissues across vertebrate groups in order to illuminate the sequential innovations that have occurred in adaptive immunity, from fish to mammal (Boehm et al. 2012).

Bony fishes or teleosts are amongst the first groups in phylogeny to possess an adaptive immune system based on the presence of molecules in the immunoglobulin superfamily. Lymphoid structures in teleost fish include thymus, spleen, the bone marrow equivalent head kidney, and various mucosa‐associated lymphoid tissues (MALT). The latter may be divided into gut‐associated lymphoid tissue, gill‐associated lymphoid tissue, the recently discovered nasopharynx‐associated lymphoid tissue and skin‐associated lymphoid tissue, but organized MALT, as found within the mucosa of endotherms, are not present in fish. The general impression is that immune cells rather form a diffuse network of leukocytes that are disseminated along the mucosal surfaces, and there are no observations of follicles as found in mammalian secondary lymphoid organs or as in the avian bursa. Thymus is the most conserved of these structures and is present in all gnathostomes, usually with a typical cortical and medullary organization. Lymph nodes are absent, and the existence of lymphatic vessels is disputed (Zapata & Amemiya, 2000; Vogel, 2010; Salinas, 2015; Flajnik, 2018). To the best of our knowledge, there are so far no reports of a bursa of Fabricius‐like structure in fish.

Important lymphoepithelium‐derived organs, including the thymus, tonsils and the bursa of Fabricius, develop from the pharyngeal pouches and the cloacal membrane where ectoderm and endoderm are juxtaposed (Varga et al. 2008; Nagy & Oláh, 2010). In our previous studies of the immune tissues of the Atlantic salmon, we focused on the pharyngeal region including the gills. These studies discovered and described interbranchial lymphoid tissue (ILT), which is strictly intra‐epithelial and lacks key thymic characteristics, suggesting an early phylogenetic emergence also of secondary lymphoid organs in this region (Haugarvoll et al. 2008; Aas et al. 2014). In the present study, we focused on the cloacal region of the Atlantic salmon. This region has previously attracted some attention with respect to the existence of abdominal pores, which is a paired set of tubular stuctures lateral to the anus and connecting the abdominal cavity with the external environment (George et al. 1982). However, lymphoid tissues or structures in this region have not been reported in fish. In the present study we show, for the first time, a structural analogue to the bursa of Fabricius in a non‐avian species. We describe a cloacal diverticulum with striking topographical similarities to the avian bursa and a prominent and dynamic lymphoepithelium that involutes around sexual maturation, just as in birds.

## Materials and methods

### Animals

Three groups of unvaccinated Atlantic salmon (*Salmo salar*) were included in the study, covering various developmental stages of the fish and the bursa. Important landmarks of salmon development are smoltification (smolt), i.e. the physiological changes needed for adaptation from freshwater to seawater, and sexual maturation. Group 1 consisted of cultivated pre‐smolt of 2–8 cm, obtained from the joint aquarium of the Norwegian Veterinary Institute and the Faculty of Veterinary Medicine, Norwegian University of Life Sciences, Oslo, Norway. Group 2 consisted of cultivated smolt of 16–20 cm, obtained from the same aquarium as Group 1, and cultivated post‐smolt fish of 49–57 cm length, obtained from the aquarium of the Norwegian Institute for Water Research, Solbergstrand, Drøbak, Norway. Group 3 consisted of wild, spawning salmon caught with a dip‐net for the river cultivation programme of River Drammenselven, Norway, and generously provided by the Sport Fishermen's Club at Hellefossen, Hokksund. Details of the different groups are presented in Table 1. Both sexes were represented in all samples. In all groups, the cloacal region with adjacent tissue was dissected and sampled. The smallest fish in Group 1 did not need dissection due to their size and were consequently sampled and sectioned whole for histology. All fish were killed in accordance with the Norwegian Aquaculture Act, §34 Euthanasia of fish.

**Table 1:** Material overview.

	Length (cm)	Origin	*n*
Group 1:pre‐smolt	2	Cultivated	22
	2.5	Cultivated	44
	4	Cultivated	29
	6–8	Cultivated	25
Group 2:smolt and post‐smolt	16–20	Cultivated	28
	49–57	Cultivated	14
Group 3:spawning adults	70–90	Wild	9

### Histological examinations

Tissue samples were fixed in 10% neutral buffered formalin for 24–48 h. This was followed by further dissection (not 2‐cm fish) to ensure sections of the bursa in horizontal, transversal and sagittal planes. The samples were then dehydrated and subsequently embedded in paraffin wax. Sections were cut 2 µm thick and mounted on glass slides, de‐waxed in xylene and progressively rehydrated in graded alcohol baths before staining with haematoxylin and eosin. Basic histological techniques were performed according to standard procedures (Bancroft & Gamble, 2008). All samples underwent basic histological examination. Further morphological investigations by immunohistochemistry and *in situ* hybridization for all antibodies and probes were conducted on at least three individuals per group for characterization of the bursal tissue, as described in detail below.

### Macroscopic investigations and radiography

**Figure 1:**
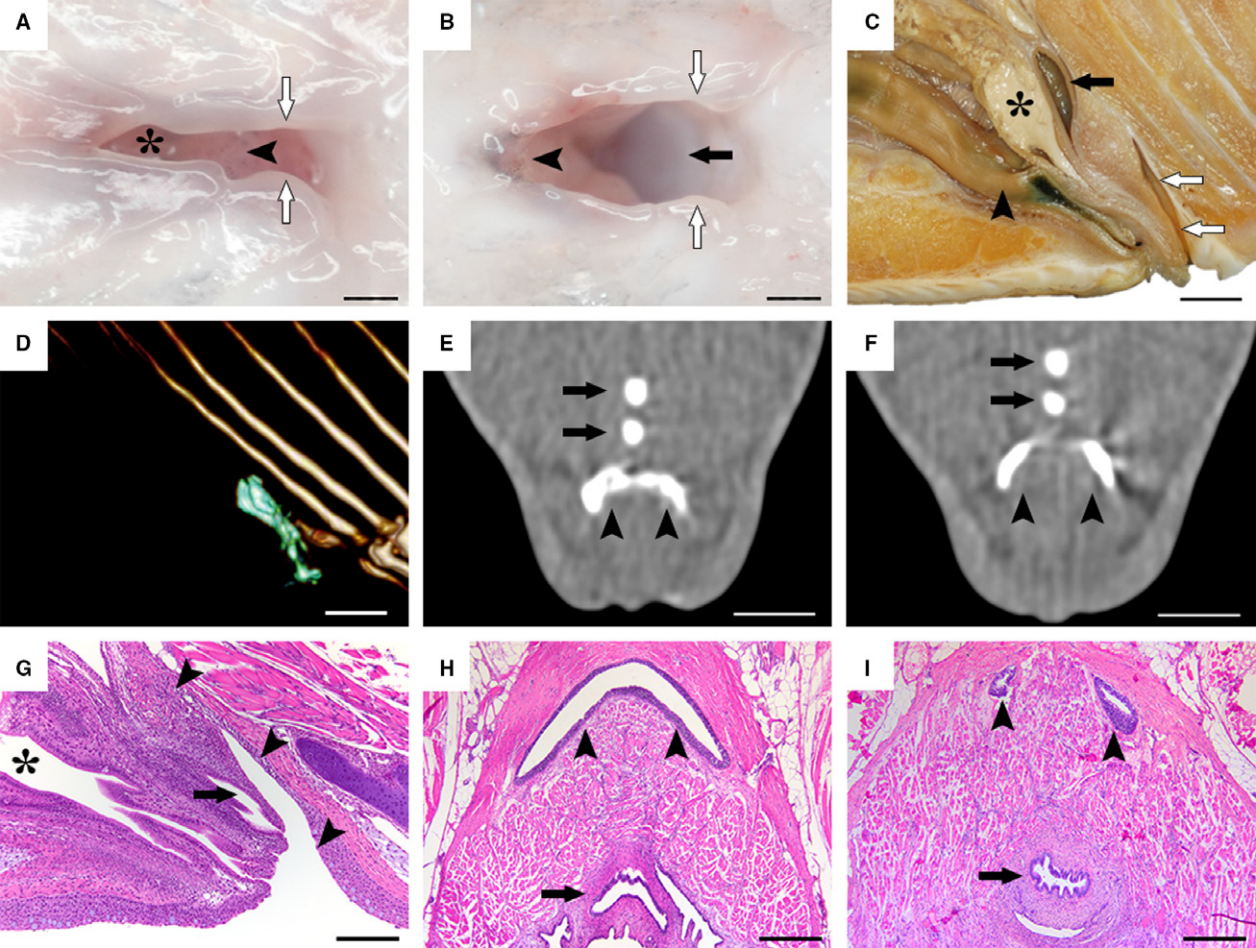
Morphological investigations of the salmon bursa. (A,B) Ventral view of the salmon cloacal region in a euthanized fish. The asterisk indicates the intestinal aperture in the cranial portion. When pulling the urogenital papilla (arrowheads) cranially and the cloacal labiae (white arrows) aside laterally, the bursa (black arrow) becomes visible. (C) Mediosaggital section of the cloacal region in a formalin‐fixed sample of a male spawning fish, showing the craniocaudal order of the hindgut (arrowhead), the genital tract (black asterisk) and the urinary tract (black arrow) which together forms the urogenital papilla, followed by the bursa (white arrows). (D–F) CT investigations with contrast fluid injected into the bursa. A three‐dimensional render (D) depicts the bursal lumen outline (green) from a lateral view. Note how the bursa (arrowheads) progresses from a crescent shape (E) into two individual blind sacs craniodorsally (F). Black arrows indicate bones of the anal fin. (G–I) Histological examination of the bursa (arrowheads) in mediosaggital (G) and horizontal (H, I) planes, reflecting that of the macroscopic (C) and computed tomography investigations (D–F), while also displaying a prominent lymphoepithelium. Black asterisk indicates the hindgut and black arrows indicate the urogenital tracts. Scale bars, 1 mm (A,B), 1 cm (C–F), 100 µm (G), 200 µm (H,I).

The images in Fig. 1A,B were captured from a recently killed fish from Group 2 in a ventral projection. For Fig. 1C, the cloacal region in a fish from Group 3 was cut mediosaggitally, fixed in formalin and photographed laterally. All images were captured using a Nikon D70s Digital single‐lens reflex camera mounted with a Nikon AF Micro‐Nikkor 60 mm f/2.8D objective (Nikon Corporation, Minato, Tokyo, Japan) and an LED Ring flash. To probe the size of the bursa and its exact topography relative to adjacent structures, one recently killed adult male salmon from Group 3 was subjected to computed tomography (CT) scanning in a dorsal position with Omnipaque 300 mg/mL (GE Healthcare, Oslo, Norway) injected into its bursal lumen for contrast. This was done using a four‐detector row CT scanner (BrightSpeed, GE Healthcare, Oslo, Norway), with a slice thickness of 1.25 and a 0.625‐mm overlap and using helical acquisition in bone and soft tissue algorithms. An OsiriX DICOM Viewer (Pixmeo SARL, Bernex, Switzerland) was used for post‐processing and capture of Fig. 1D and Video S1, while CARESTREAM Vue PACS (Carestream Health, Rochester, NY, USA) was used for Fig. 1E,F and Video S2.

### Immunohistochemistry

We used a commercial monoclonal antibody to detect cytokeratin in epithelial cells (ab27988; Abcam, Cambridge, UK; diluted 1 : 50). To detect T cells and antigen‐presenting cells, we used monoclonal antibodies for CD3ε (Boardman et al. 2012) and major histocompatibility complex (MHC) class II (Hetland et al. 2010) in dilutions 1 : 400. The use of all antibodies followed the same staining procedure as described below. Steps were performed at room temperature unless otherwise stated. Sections were cut 4 µm thick and mounted on positively charged glass slides (Superfrost^©^; Mentzel, Braunshweig, Germany), incubated for 24 h at 37 °C and thereafter for 30 min at 58 °C, de‐waxed in xylene and progressively rehydrated in graded alcohol baths before transfer to distilled water. Sections were subsequently demasked by autoclavation in a 0.01‐m citrate buffer, pH 6.0 at 120 °C for 10 min. Sections were rinsed three times in phosphate‐buffered saline (PBS) prior to treatment with phenylhydrazine (0.05%; Sigma‐Aldrich, St Louis, MO, USA) for 40 min at 37 °C incubation to inhibit endogenous peroxidase, followed by yet another 3× rinse in PBS. Non‐specific binding was prevented by treatment with normal goat serum, diluted 1 : 50 in tris‐buffered saline (TBS) with 5% bovine serum albumin (BSA) for 20 min. The blocking solution was removed and the sections incubated with the primary antibody, diluted in TBS with 1% BSA, for 30 min. Sections were rinsed three times with TBS and incubated again with a secondary antibody (EnVision^©^ System kit; Dako, Glosrup, Denmark) for 30 min, following yet another 3× rinse with TBS. To evoke a red or brown colour respectively, the sections were treated with amino ethyl carbazol for 14 min or 3,3′Diaminobenzidine for 7 min (EnVision^©^ System kit). Sections were then washed with distilled water and counterstained with Mayer’s haematoxylin for 1.5 min and mounted with Aquatex^®^ mounting medium (Merck KGaA, Darmstadt, Germany). Negative controls were performed without primary antibody and positive controls were performed on skin (for cytokeratin) and head kidney (for CD3 and MHC class II).

### *In situ* hybridization

RNAscope^®^ 2.5 HD Assay‐red (Advanced Cell Diagnostics, Newark, CA, USA) was used for all *in situ* hybridization procedures and according to the manufacturer’s instructions (Wang et al. 2012). All steps were performed at room temperature unless otherwise stated. In short, paraffin‐embedded tissue sections of 4 µm, mounted on positively charged glass slides (Superfrost^©^; Mentzel) and air‐dried for at least 24 h, were incubated at 60 °C for 90 min with subsequent 2 × 5 min xylene and 2 × 1 min 100% ethanol treatment for dewaxing. Samples were then treated with hydrogen peroxide for 10 min to block endogenous peroxidase. Pretreatment included heat treatment in RNAscope^®^ Target Retrieval Reagent at 100 °C for 15 min and protease treatment for 10 min at 40 °C to permeabilize the cells. For hybridization, slides were incubated with target ZZ‐probe solution for 2 h at 40 °C. After hybridization, the signal was amplified by sequentially incubating the slides with the six amplification solutions provided with the assay kit. Signal detection was performed by incubation with Fast Red chromogenic substrate for 10 min and counterstaining by incubation with a 50% Gill’s haematoxylin solution for 2 min. The samples were then mounted with EcoMount (BioCare Medical, Pacheco, CA, USA). All probes were designed and produced by the manufacturer based on user‐provided sequences, and have been catalogued and made commercially available. Details regarding the probes and positive and negative control probes including gene, target region, accession number and the manufacturer’s catalogue number are available in Table 2. As an additional verification of the specificity, all probes were screened against salmon liver, head kidney and lateral skin as control tissues, sampled from individuals in Group 2.

**Table 2:** Target and control probes for *in situ* hybridization.

Probe	Accession no.	Target region, bp	Catalogue no.
Target			
IgT	GQ907003	3–883	532171
IgM	XM_014203125	219–1157	532181
IgD	AF141607	116–1123	575881
TCR α‐chain	AY552002	402–718	558651
TCR δ‐chain	XM_014140004	216–693	558661
CCL19	XM_014128861	134–444	558671
AID	XM_014151382	62–671	562621
RAG1	XM_014125106	1302–2131	572671
RAG2	XM_014125109	544–1597	572681
Control			
DapB (negative)	EF191515	414–862	310043
PPIB (positive)	NM_001140870	20–934	494421

### Scanning electron microscopy

The cloacal region of one fish from Group 2 sectioned horizontally was used for the scanning electron microscopy analysis. The sample was first fixed in a mixture of 1.25% glutaraldehyde and 2% paraformaldehyde dissolved in 0.1 m cacodylate buffer, pH 7.2. Subsequently, the sample was washed in PBS buffer to remove the fixative prior to dehydration in increasing ethanol concentrations. The sample was then critical point dried, mounted on a stub and sputter coated with gold. The analysis was conducted on a Zeiss EVO 50 EP scanning electron microscope (Carl Zeiss AG, Oberkochen, Germany).

## Results

### Morphological examination of the bursal structure

**Figure 2:**
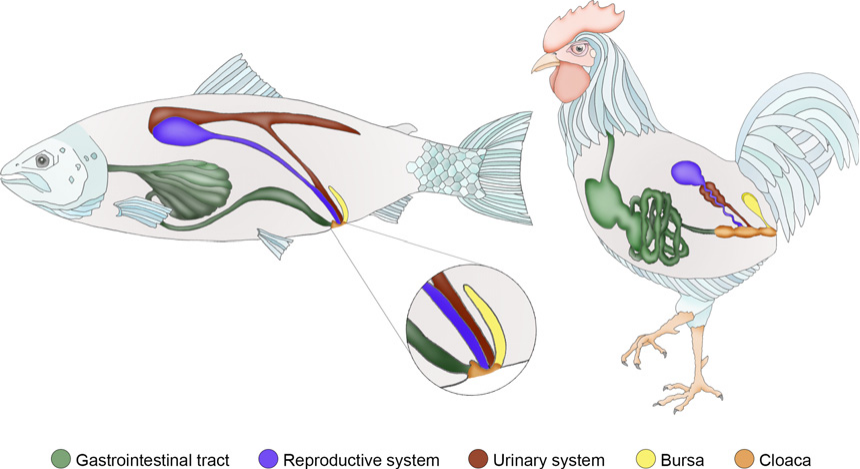
Schematic illustrations comparing the cloacal region of Atlantic salmon and birds, elucidating the similar craniocaudal order of the intestinal, genital, urinary, and finally the bursal aperture in both salmon and bird. These apertures terminate in a joint compartment (cloaca), extensively developed in birds, but only rudimentary in salmon.

Initial macroscopic investigations of the salmon cloacal region revealed the salmon bursa as a diverticulum caudal to the urogenital papilla (Fig. 1A–C). Furthermore, CT investigations revealed the bursa to extend craniodorsally in a crescent shape along the caudal rim of the urogenital papilla and terminating distally in a right and left ventricle or blind sac (Fig. 1D–F, Video S1 and S2). This was confirmed by histological sections of the bursa, which revealed a prominent epithelium continuous with the epidermis surrounding the cloaca as well as the urogenital and intestinal aperture (Fig. 1G–I). The cloacal bursa was invariably present in all individuals and in both sexes (as is also the case in birds). These investigations allowed us to revise the gross anatomy of the Atlantic salmon cloacal region and compare it to that of birds, elucidating conspicuous similarities (Fig. 2).

### Characterization of the bursal epithelium

**Figure 3:**
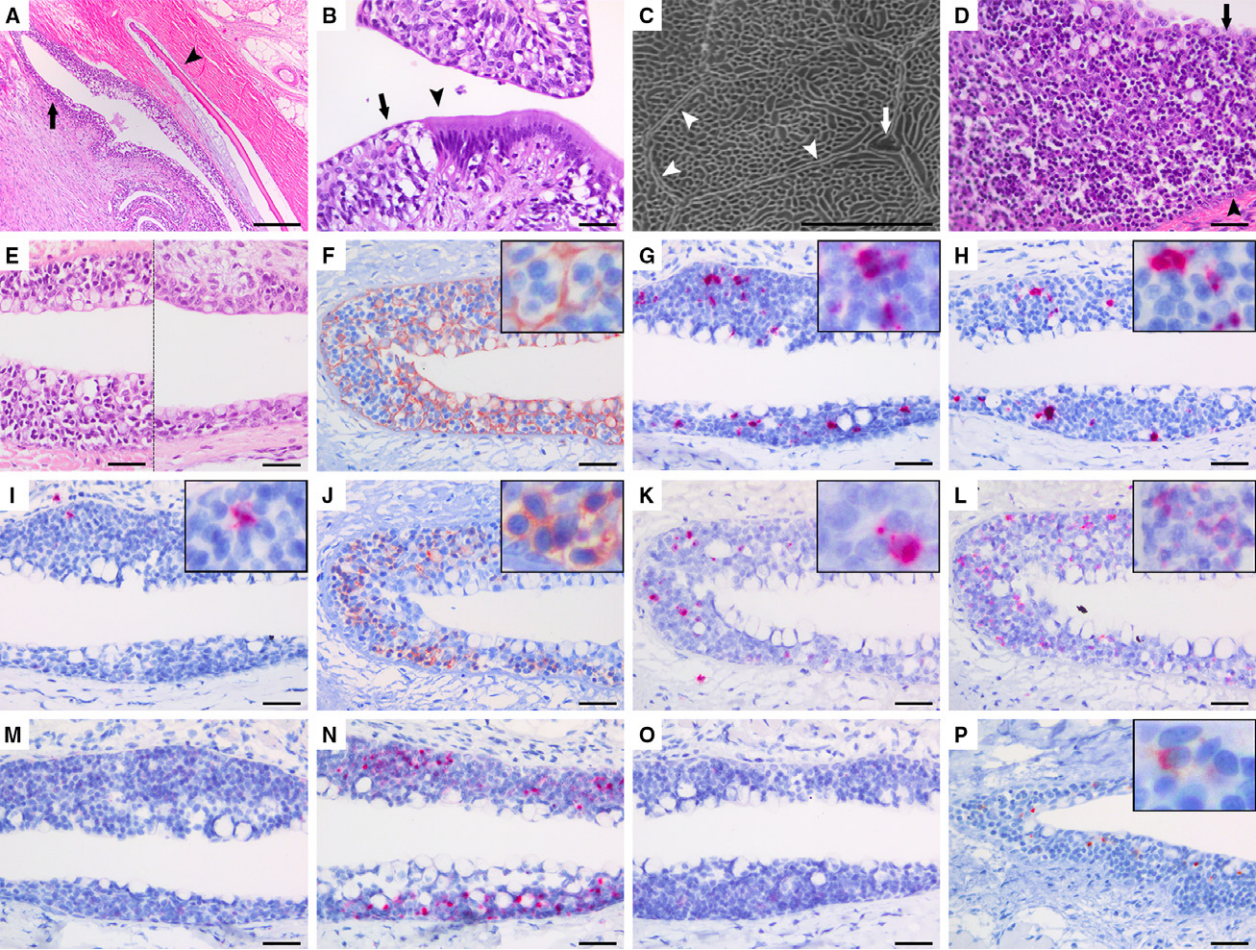
Histological investigations of the salmon bursa lymphoid tissue. (A) Lateral view of a sagittal section of a fish in an early developmental stage (Group 1), showing the placement of a dermal scale (arrowhead) beneath the bursal epithelium (black arrow) in a similar arrangement as in truncal skin. (B) Section from the salmon cloacal region demonstrating the epithelial transition between the ectodermal epidermis (black arrow) and the endodermal intestinal mucosa (arrowhead). (C) Scanning electron micrograph of the epithelial surface in the salmon bursa (Group 2) demonstrating a fingerprint‐like microridged pattern characteristic of fish skin epidermis. The gross outline of an individual epithelial cell is indicated (arrowheads) in addition to a mucus cell pore (arrow). (D) Large quantities of lymphocytes with small, dense and spherical nuclei, interspersed within reticulated epithelial cells in a bursal terminal blind sac where the epithelium is most developed (Group 2). Epithelial surface (arrow) and basal membrane (arrowhead) are marked. (E) The lateral parts of the bursa (left side of the image) contains a thicker epithelium that is richer in lymphocytes compared to the medial part (right side of the image). Both halves of the image are captured from the same horizontal section. (F) Cytokeratin‐positive epithelial trabeculae form a meshwork containing lymphocytes (cytokeratin immunostain, haematoxylin counterstain). (G–I) *In situ* hybridization for immunoglobulin transcripts show several IgD^+^ (G) and IgM^+^ (H) lymphocytes, but scarce amounts of IgT^+^ B cells (I). (J) CD3^+^ cells are abundant throughout the epithelium (CD3 immunostain, haematoxylin counterstain). Both γ/δ (K) and α/β T cells (L) are present in the bursal lymphoepithelium, but the latter is the most numerous (*in situ* hybridization). (M) *In situ* hybridization for activation‐induced cytidine deaminase (AID) shows no positive transcripts in the bursal epithelium. (N) The bursal lymphoepithelium is positive for RAG‐1 transcripts (*in situ* hybridization). (O) No RAG‐2 transcripts are detected (*in situ* hybridization). (P), Several major histocompatibility complex (MHC) class II^+^ cells are present within the bursal lymphoepithelium (MHC class II immunostain, haematoxylin counterstain). Scale bars, 100 µm (A), 20 µm (B), 5 µm (C), 20 µm (D–P).

The bursal epithelium resembled the stratified squamous epithelium of the epidermis much more so than the columnar epithelium of the intestinal mucosa, and early developmental stages contained dermal scales in close proximity to the bursa (Group 1, Fig. 3A). In addition, there was no clear transition from epidermal to bursal epithelium, as was the case between epidermis and intestinal mucosa (Fig. 3B). Scanning electron microscopy revealed that the bursal epithelial cells also displayed a fingerprint‐like microridged pattern on their surface (Fig. 3C). Mid‐sized fish (Group 2) exhibited substantial quantities of lymphocyte‐like cells within the epithelium (Fig. 3D). Additionally, the craniolateral parts of the bursa generally displayed a thicker epithelium, containing more lymphocytes than the medial part (Fig. 3E). Cytokeratin immunostaining of fish from Group 2 revealed a layering of the bursal epithelium, where mucus cells and attenuated epithelial cells covered a reticulated epithelium. Here, transformed epithelial cells projected interconnecting cytoplasmic trabeculae, forming a meshwork of intraepithelial pockets where aggregates of lymphocytes could be seen (Fig. 3F).

*In situ* hybridization for Ig‐transcripts displayed several IgD^+^ and some IgM^+^ B cells. Only occasional IgT^+^ cells were found, which is a teleost functional analogue to IgA^+^ B cells in mammals (Fig. 3G–I). However, when labelling with antibodies raised against the pan T‐cell marker CD3, we detected strong signals throughout the epithelium (Fig. 3J). *In situ* hybridization for γ/δ and α/β T cells revealed that both types were present, although the latter appeared more numerous (Fig. 3K,L). We did not detect activation‐induced cytidine deaminase (AID) transcripts within the bursal lymphoepithelium in any of the groups (Fig. 3M). The bursal epithelium was positive for RAG‐1 by *in situ* hybridization, but not RAG‐2 (Fig. 3N,O). Immunostaining for MHC class II displayed several positive cells within the bursal epithelium (Fig. 3P). Positive and negative controls for immunohistochemistry and *in situ* hybridization tested positive and negative, respectively (data not shown).

### Epithelial variations between developmental stages

**Figure 4:**
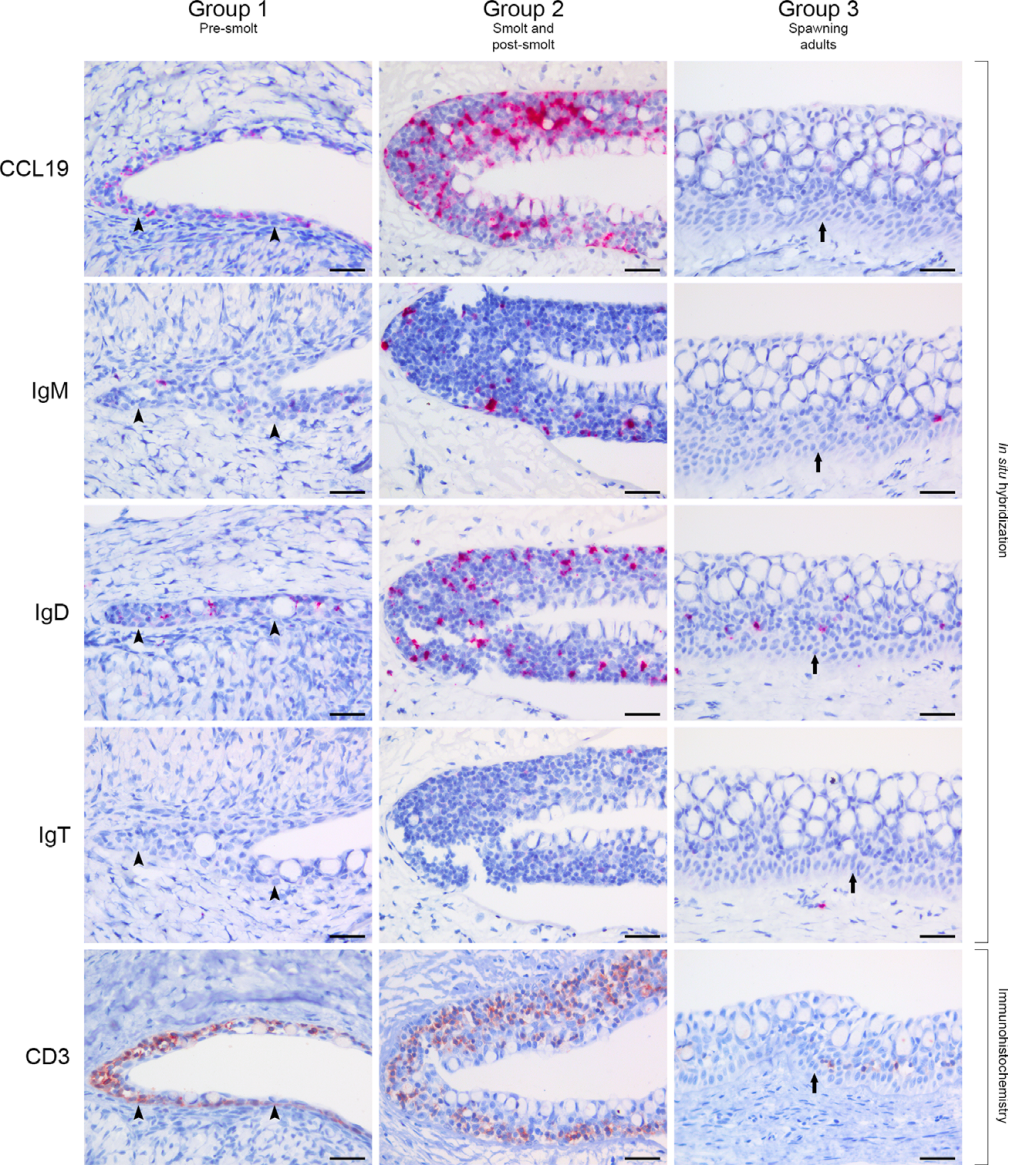
Comparison of bursal lymphoepithelium between different developmental stages of salmon. The groups described are presented in Table 1. Group 1: the thin bursal epithelium is positive for CCL19 transcripts (*in situ* hybridization). Occasional IgM^+^ and IgD^+^ B cells (*in situ* hybridization), as well as several CD3^+^ T cells (CD3 immunostain, haematoxylin counterstain) are seen at this stage. IgT transcripts (*in situ* hybridization) were not detected. Arrowheads indicate the basal membrane. Group 2: a more advanced and reticulated bursal epithelium is highly positive for CCL19. Several IgM^+^, many IgD^+^, occasional IgT^+^ and abundant CD3^+^ lymphocytes are embedded within epithelial meshwork niches. Group 3: the bursal lymphoepithelium has regressed and is structurally similar to cutaneous epidermis, with a prominent *stratum basale* (arrow) and several mucus cells. CCL19 transcripts are scarce, but occasional lymphocytes are still present, with IgD^+^ B cells and CD3^+^ T cells being the most common. Scale bars, 20 µm.

We then investigated the development of the salmon bursal lymphoepithelium by screening different developmental stages with markers for the chemokine CCL19 and various lymphocytes. In Group 1, we observed individual variation with respect to the presence of positive cells, but both T and B cells were detectable (Fig. 4; Group 1). Reticulated epithelial cells were not identified at this early stage, and the bursal epithelium appeared more as an ordinary cutaneous epidermis. In Group 2, a prominent lymphoepithelium highly positive for CCL19 and with a lymphocyte content as described in the previous paragraph was present in all investigated individuals (Fig. 4; Group 2). Following sexual maturation, CCL19 transcripts and subsequently the amount of T and B cells decreased dramatically, and the epithelium yet again resembled that of the cutaneous epidermis (Fig. 4; Group 3).

### Investigations of control tissue for *in situ* hybridization probes

**Figure 5:**
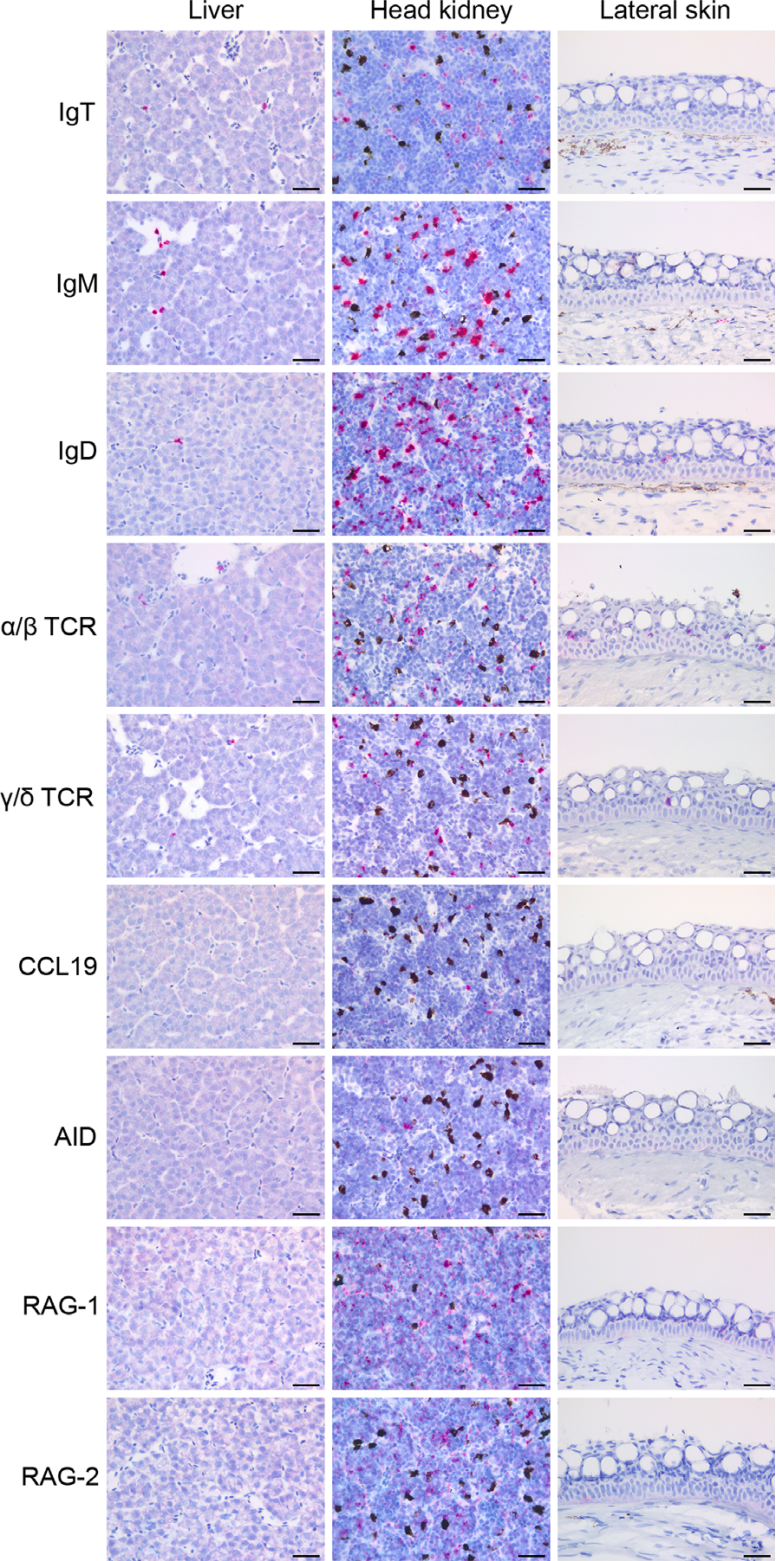
Screening of control tissues with probes used for *in situ* hybridization. Liver: hepatocytes are negative for all probes as expected. Note the positive cells (red) for the lymphocyte markers (IgT, IgM, IgD, α/β and γ/δ), all within blood vessels or sinusoids. Head kidney:the bone marrow equivalent organ in fish, functioning as a primary and secondary lymphoid organ. All probes provided positive signals (red). The black colouring is melanin confined to melano‐macrophages. Lateral skin, Sections reveal positive transcripts for α/β and γ/δ TCR as well as IgD within the epithelium, with α/β being the most numerous. Transcripts for IgM and IgT are scarce in the epidermis, however, some positivity for IgM is seen within the underlying dermis. Probes for CCL19 and activation‐induced cytidine deaminase (AID) transcripts are both negative. Occasional transcripts are detected for RAG‐1, but not for RAG‐2. Scale bars, 20 µm.

The liver parenchyma was negative for all probes, but occasional transcripts for IgT, IgM and IgD B cells, as well as α/β and γ/δ T cells, were detected within blood vessels or sinusoids (Fig. 5, liver). In head kidney, all probes were positive and the respective cells exhibited an expected morphology and distribution (Fig. 5, head kidney). Lateral skin sections contained scarce amounts of B cells, and IgD^+^ cells were the most frequent subtype. Several α/β T cells, but few γ/δ T cells were present. Probes for CCL19 and AID were both negative. We detected very scarce amounts of RAG‐1 transcripts, but none for RAG‐2 (Fig. 5; lateral skin).

## Discussion

In the present study, we examined the salmon cloacal region and discovered a substantial lymphoepithelial tissue in a previously undescribed bursa, situated caudal to the urogenital papilla and thus analogous to the anatomical placement of the bursa of Fabricius in birds (Nagy & Oláh, 2010). It is highly conspicuous that such a large structure has remained undescribed; however, a comprehensive review of the literature on fish anatomy revealed no reports of a cloacal bursa in any teleost species. We thus undertook a careful anatomical examination of the site. Although teleosts do not possess a true cloaca of the avian type, two shielding epithelial folds or longitudinal labiae cover the orifices of the intestinal, reproductive and urinary tracts in salmonids (George et al. 1982). This forms a joint compartment of endodermal and ectodermal juxtaposition, similar to the proctodeum of the avian cloaca. Recently, Nagy & Oláh (2010) demonstrated experimentally that the epithelial primordium of the bursa of Fabricius is of ectodermal origin, developing from the anal invagination that forms the proctodeum. Our results strongly indicate that the epithelial anlage of the salmon bursa shares this origin, and we suggest a similar organogenesis in both fish and birds.

In the bursa of Fabricius, follicles of B cells dominate the histological appearance, but T cells are also present (Ciriaco et al. 2003; Ekino & Sonoda, 2014). However, germinal centres have not been described in teleosts (Flajnik, 2018). In the presently reported structure, lymphocytes were dispersed throughout the salmon bursal epithelium. This appears to be the principle organization of MALT in teleost fish (Salinas, 2015). Interestingly, the terminal ventricles of the salmon bursa appeared to be the most developed, and the craniolateral parts on both sides in general richer in lymphocytes than the medial part. A similar biased distribution is also seen in early developmental stages in the avian bursal epithelium, where the cranial portion receives more lymphocytes than the caudal portion (Nagy et al. 2004; Nagy & Oláh, 2010).

The reported lymphocytes were compartmentalized in a three‐dimensional meshwork constituted by reticulated epithelial cells. This composition is classified as lymphoepithelial tissue, also used to describe the epithelium lining the crypts of mammalian tonsils and that of the avian bursa of Fabricius (Oláh & Glick, 1992; Perry & Whyte, 1998). In fish, lymphoepithelium has previously been described in the subcapsular zone of the thymus in addition to the ILT in gills (Chilmonczyk, 1983; Koppang et al. 2010; Bjørgen et al. 2019). The organization of such a lymphatic niche forms an important microenvironment, in which the scaffolding epithelial cells facilitate lymphocyte differentiation and maturation (Capece & Kim, 2016). Considering the proposed analogy, we would expect the majority of the observed lymphocytes to be B cells, but as our results show, this did not turn out to be the case. In our investigations, we detected several IgD and IgM transcripts, but scarce amounts of IgT. Interestingly, this demonstrates a different situation compared to the skin epithelium, where IgT^+^ B lymphocytes are the major B‐cell subset (Xu et al. 2013). As in mammals, the functions of teleost IgD is still largely unknown, but is believed co‐expressed with IgM in naïve B cells and downregulated post activation (Edholm et al. 2011). IgD/IgM double‐positive cells are thought to be present in all examined teleost species, and supposedly represent the majority of B cells in fish (Diáz‐Rosales et al. 2019). It seems plausible then that IgD^+^/IgM^+^ B cells can encounter antigens and activate in the salmon bursa. We did not determine double positivity, however, and cannot rule out that the reported IgD^+^ and IgM^+^ B cells are different populations. This will be a subject for future study. Interestingly, a large majority of the salmon bursal lymphocytes were immunopositive for the pan T‐cell marker CD3. Further investigations revealed that both γ/δ and α/β T cells were present, the latter being the most numerous. Contrary to the bursa of Fabricius, T cells seem to be the major infiltrating lymphocyte population in the salmon bursa.

Because the bursa of Fabricius is paramount to birds as both a primary and secondary lymphoid organ, we wanted to investigate whether the salmon bursa fulfills a similar purpose. In mammals, V(D)J recombination by co‐expression of RAG‐1 and RAG‐2 genes is the main diversifier of the lymphocyte repertoire. This is not the case in birds, however, where traditional Ig gene rearrangements, although still present and indistinguishable in function, do not generate the same amount of diversity as in mammals (Ratcliffe & Jacobsen, 1994). Rather, such rearrangements provide a substrate for further diversification of B cells via Ig gene conversion, where upstream pseudogenes of the Ig variable (V) regions replace homologues in the already rearranged gene (Ratcliffe & Jacobsen, 1994). Gene conversion is dependent on the mutator AID, which is highly expressed in the bursa of Fabricius (Arakawa et al. 2002). In addition, this enzyme facilitates class‐switch recombination and somatic hypermutation (SHM). The latter is known to occur in teleost fish (Bilal et al. 2018), but it is not yet fully understood, partly because germinal centres are lacking. While generally being regarded as a driver of the secondary repertoire through affinity maturation, some evidence suggests SHM is also a means of diversifying the primary repertoire, namely that of Ig and TCR γ (Barreto & Magor, 2011). Hitherto, AID expression in teleosts is found in relation to melano‐macrophage centres in both the bone marrow equivalent head kidney and in the spleen, in addition to some individual cells in the intestine (Saunders et al. 2010). Considering the abundance of T and B cells in the salmon bursa, it could very well be a site for AID‐induced somatic editing, e.g. affinity maturation by SHM; however, we did not detect AID transcripts within the bursal lymphoepithelium in any of the groups. Furthermore, we aimed to investigate whether recombination activity occurred in the salmon bursa. Lymphoepithelial positivity for RAG‐1, but not RAG‐2, indicates that Ig gene rearrangements are unlikely to take place. Together, these findings do not suggest any primary lymphatic function of the salmon bursa in contrast to the avian bursa of Fabricius.

Analogous to the situation in birds, the location of the salmon bursa is ideal for sampling and presentation of enteric and environmental antigens, an important precedent for priming of naïve lymphocytes. While B cells can encounter and bind to antigens directly through their Ig‐receptor, T cells depend on presentation of antigen through MHC molecules for activation. We found several MHC class II^+^ cells within the reticulated bursal lymphoepithelium of Group 2, the developmental stages where the lymphoepithelium appears most advanced and external labiae effectively cover the apertures of the cloacal region, forming a chamber resembling the avian cloaca. This likely helps trap antigen within the bursal lumen as is the case in birds (Ekino & Sonoda, 2014), furthering the possibility of lymphocyte activation and ensuring an adept immune response, whether this induces tolerance or immunization. Future functional studies should address if such mechanisms are present in the salmon bursa.

The development and subsequent regression of the salmon bursal lymphoid tissue following sexual maturation must be accentuated. This is a trait so far observed only in the primary lymphoid organs thymus and bursa of Fabricius. In birds, the primordial bursal epithelium undergoes follicle formation and transforms into lymphoepithelial tissue by infiltration of hematopoietic cells between embryonic days 11 and 14 (Nagy & Oláh, 2010). Subsequently, it involutes around the time of sexual maturation (Ciriaco et al. 2003). We have shown that a thin epithelial anlage in newly hatched pre‐smolt fish (Group 1) with few lymphocytes transformed into a well‐developed lymphoepithelium in post‐smolts (Group 2). In sexually mature fish (Group 3), the lymphoepithelium had regressed and the epithelium was nearly devoid of lymphocytes. Importantly, the presence of lymphocytes coincided with epithelial positivity for the cytokine CCL19, similar to the teleost reticulated lymphoepithelium in the thymic subcapsular zone and in the ILT (Bjørgen et al. 2019). This chemoattractant is important for trafficking of both B and T lymphocytes, and its expression in mammals is restricted to primary and secondary lymphoid organs (Comerford et al. 2013). Together, these findings demonstrate an age‐dependent development followed by involution of the lymphoepithelial tissue, similar to the thymus and the avian bursa of Fabricius.

In conclusion, our results reveal that an undescribed lymphoepithelial compartment with striking anatomical and developmental similarities to the bursa of Fabricius is present in the cloacal region of the Atlantic salmon. This raises the possibility of an earlier phylogenetic diversification of a lymphoid organ at this site than previously thought, or alternatively, a convergent evolutionary event. Further speculations regarding the structure’s origin must however cease until additional species are investigated and functional studies have been undertaken. The findings presented in this paper provide a basis for such future studies, furthering our knowledge and understanding of fish mucosal immunity and evolutionary immunology.

## Author contributions

O.M.L. discovered the salmon bursa. O.M.L., H.B., I.H., and E.O.K. designed the experiments. H.B. was responsible for the *in situ* hybridization experiments. I.H. was responsible for probe target identification for the different genes. O.M.L. and E.O.K. wrote the manuscript. All authors participated in data analysis and commented on the manuscript.

## Conflict of interest

The authors declare no competing interests.

## Supporting information


**Video S1.** Three‐dimensional modelling of the salmon bursa by CT scanning.

**Video S2.** Serial CT images of the salmon bursa injected with contrast fluid.
