# Supplementary material for: A teleost structural analogue to the avian bursa of Fabricius
Source: J Anat. 2019 Dec 26;236(5):798–808. doi: 10.1111/joa.13147 (PMC7163591; doi:10.1111/joa.13147)
Supplement: Supplementary file 3 [file JOA-236-798-s003.docx]

**Supporting Information**

**Video S1** Three-dimensional modelling of the salmon bursa by CT-scanning. Contrast fluid injected into the bursal lumen can be seen cranial to the bones of the anal fin (see also green outline in Fig. 1d). Pelvic and dorsal fins were removed in post-processing of the image to improve visibility.

**Video S2** Serial CT images of the salmon bursa injected with contrast fluid. Located ventrally, the bursa is first visible as a circular lumen, progressing into a crescent shape, curving around the caudal edge of the urogenital papilla, before terminating craniodorsally in a left and right sac, as also depicted in Fig. 1e, f.
